# An integrated phenotypic and molecular approach to identify salinity-tolerant lentil (*Lens culinaris* Medik) genotypes

**DOI:** 10.3389/fgene.2026.1863296

**Published:** 2026-06-23

**Authors:** Ashutosh Kumar Yadav, Narayana Bhat Devate, Surendra Barpete, Vijayata Singh, Srilekha Basarahalli, Arpita Das, Berhane S. Gebregziabher, Jitendra Patidar, Rohit Namdeo, Shiv Kumar

**Affiliations:** 1 Food Legume Research Platform (FLRP) of International Center for Agricultural Research in the Dry Areas (ICARDA), Amlaha, Madhya Pradesh, India; 2 ICAR-Central Soil Salinity Research Institute, Karnal, India; 3 Bidhan Chandra Krishi Viswavidyalaya, Nadia, West Bengal, India; 4 Crop Genetics and Breeding, Melkassa Agricultural Research Center, Ethiopian Institute of Agricultural Research, Addis Ababa, Ethiopia

**Keywords:** cumulative stress tolerance index, gene expression, lentil, morpho-physiological traits, salinity stress

## Abstract

Salinity in soil and irrigation water has emerged as a serious challenge to global agricultural productivity. Lentil (*Lens culinaris* Medik.) is highly sensitive to salinity, particularly during early seedling establishment, which adversely affects growth and development. Systematic screening at the seedling stage can therefore serve as an effective strategy to identify novel sources of salt-tolerant donors. In this study, 50 elite breeding genotypes from ICARDA breeding program were evaluated for seedling-stage salinity tolerance under salt stress (electrical conductivity of 8 dS m^−1^) and control conditions through pot culture. Important morpho-physiological traits, including shoot and root length and their biomass were recorded at 30 days after sowing, revealing substantial genetic variability. Based on the cumulative salt tolerance index (CSTI), 35 genotypes were classified as salt sensitive, eight as moderately tolerant, and seven as salt tolerant, with “*X2018-482-S5*” as the most tolerant and “*FLIP2014-083L-S9*” as the most sensitive. Selected tolerant genotypes were further evaluated under saline field conditions, where X2018-482-S5, ILL7547, X2011S-89-23-S1, X2011S-91-77-2-S1, and ILL6819/ILL6207-S2 exhibited stable growth and yield performance comparable to the tolerant checks PSL9 and PDL1. Gene expression analysis revealed coordinated upregulation of the *High-Affinity Potassium Transporter* (HKT) and *NAC domain-containing protein-72* in tolerant genotypes, with significantly higher transcript levels compared to sensitive check FLIP2014-083L-S9 at 6 and 12 h post treatment with 100 mM NaCl treatment, indicating enhanced ion homeostasis and stress-responsive regulation. Overall, the integration of seedling-stage phenotyping, field validation, and molecular analysis provides robust evidence for identifying promising salinity tolerant lentil genotypes for breeding programs targeting salt-affected environments.

## Introduction

Soil salinity represents one of the most serious abiotic constraints limiting agricultural productivity worldwide, particularly in arid and semi-arid agro-ecosystems. Its incidence is increasing rapidly due to climate change, poor irrigation management, inadequate drainage, secondary salinization, and the rise of saline groundwater tables (Kokten et al., 2010). Moreover, the indiscriminate use of brackish irrigation water coupled with high evaporation and insufficient salt leaching accelerates the accumulation of soluble salts in the soil profile, ultimately deteriorating soil health and reducing crop productivity (Kumar and Sharma, 2020). Globally, more than 800 million hectares of land are affected by salinity and sodicity, accounting for a substantial proportion of irrigated agricultural areas (Shrivastava and Kumar, 2015; Machado and Serralheiro, 2017). In India alone, nearly 6.7 million hectares are salt-affected, posing a significant threat to crop production and food security (Kumar and Sharma, 2020; Neha et al., 2022). Saline soils, characterized by elevated concentrations of soluble salts, primarily Na^+^ with chloride and sulfate anions with electrical conductivity exceeding 4 dS m^−1^, impose severe constraints on plant growth and productivity. These effects are mediated through osmotic stress and ion toxicity, which collectively disrupt plant growth, water uptake, nutrient balance, cellular homeostasis, and ultimately culminate in significant yield losses (Munns and Tester, 2008).

The physiological impact of salinity affects plants in two sequential phases. The initial osmotic phase occurs immediately after exposure to salts, restricts water availability, thereby inhibiting cell expansion and shoot growth, while the subsequent ionic phase results from excessive accumulation of toxic ions, particularly Na^+^ and Cl^−−^, in plant tissues, leading to premature senescence and metabolic dysfunction (Munns and Tester, 2008; Liang et al., 2024; Shabala and Munns, 2017). At the cellular level, elevated Na^+^ concentration interferes with K^+^ uptake, disrupts ion homeostasis, inhibits enzyme activity, and impairs photosynthesis and protein synthesis (Wakeel, 2013; Flowers et al., 2015) In addition, salinity induces overproduction of reactive oxygen species (ROS), leading to oxidative damage through lipid peroxidation, membrane damage, and degradation of cellular components (Farooq et al., 2017). To mitigate these deleterious effects, plants activate antioxidant defense systems and accumulate compatible solutes such as proline and soluble sugars, which facilitate osmotic adjustment and protect cellular integrity (Hasanuzzaman and Fujita, 2022).

Grain legumes are inherently sensitive to salinity stress, despite their pivotal role in sustainable agriculture and nutritional security. Lentil (*Lens culinaris* Medik.) is an important cool-season legume crop valued for its high protein content, essential micronutrients, and contribution to soil fertility through biological nitrogen fixation. It is widely cultivated across South Asia, West Asia, North Africa, and parts of temperate regions, where salinity frequently limits productivity (Kumar et al., 2015). Compared to many other legumes lentil is particularly susceptible to salinity, exhibiting substantial reductions in germination, seedling establishment, and yield even at relatively low soil electrical conductivity levels (Jayasundara et al., 1997; Katerji et al., 2001; Kokten et al., 2010). Yield losses can range from 50% to over 90%, depending on stress intensity, growth stage, and genotype (Singh et al., 2017; Kumawat et al., 2017).

Salinity stress adversely affects germination, seedling establishment, root and shoot growth, photosynthetic efficiency, and reproductive development in lentil (Foti et al., 2019; Panuccio et al., 2022). These effects are closely associated with disrupted ion homeostasis, particularly reduced K^+^/Na^+^ ratio, impaired water relations, and enhanced oxidative damage. To counteract these effects, plants employ an orchestra of mechanisms, including Na^+^ exclusion, vacuolar sequestration *via* Na^+^/H^+^ antiporters, and regulation of long-distance ion transport through specialized transport proteins (Horie et al., 2012; Farooq et al., 2017). High-Affinity Potassium Transporters (HKT) play a pivotal role in retrieving Na^+^ from the xylem, thereby limiting its translocation to aerial tissues and maintaining ionic balance (Roy et al., 2014). Root traits also play a crucial role in regulating ion uptake and water absorption under saline conditions (Singh et al., 2017), yet these aspects remain comparatively underexplored in lentil. At the molecular level, salinity tolerance is regulated by intricate regulatory networks involving stress-responsive genes and transcription factors. Among these, NAC (NAM, ATAF, and CUC) proteins act as key regulators by modulating the expression of downstream genes associated with osmotic adjustment, antioxidant defense, and cellular protection (Shao et al., 2015; Zhu, 2016). In lentil, recent genomic and transcriptomic studies have identified both HKT transporters and NAC domain-containing proteins as critical determinants of salinity tolerance, contributing to improved ion regulation and enhanced stress resilience (Dissanayake et al., 2021; Singh S. et al., 2021).

Substantial genetic variability for salinity tolerance has been reported within lentil germplasm, including landraces, cultivated varieties, and wild relatives. Previous studies have employed hydroponic, pot, and field-based screening approaches to identify salt-tolerant genotypes using morpho-physiological, biochemical, and yield-related traits (Singh et al., 2017; Dissanayake et al., 2020; Shekhawat et al., 2025). Screening at the seedling stage is particularly advantageous due to its higher sensitivity to salinity and ease of phenotyping, although validation under field conditions is essential to ensure stability of tolerance (Gregorio et al., 1997; Singh et al., 2017). Despite these efforts, many studies have relied either on controlled environments or on a limited set of traits, leaving gaps in understanding genotype performance across contrasting growing conditions.

Integrated evaluation of lentil genotypes that combines controlled environment screening, field validation along with molecular characterization is therefore critical to identify stable and agronomically relevant sources of salinity tolerance. Such strategies enable a more realistic assessment of genotype × environment (GE) interactions and facilitate to elucidate tolerance mechanisms that can be exploited in breeding programs.

In this context, the present study aimed to screen a diverse set of 50 lentil elite breeding lines under both pot and field conditions to characterize their responses to salinity stress. The specific objectives were to (i) quantify the effects of salinity on morpho-physiological, and yield-related traits, (ii) identify stable salt-tolerant genotypes across contrasting environments, and (iii) elucidate the role of key candidate genes associated with ion homeostasis and stress response.

## Materials and methods

### Plant material

The present study included 50 lentil genotypes comprise of advanced breeding lines developed by ICARDA using elite breeding materials, germplasm accessions, and landrace collections at the ICARDA breeding station in Morocco, along with two salt-tolerant checks, namely PDL 1 and PSL 9. The pedigree details and unique identifiers of these lines have been provided in Table 4, which allow the origin and breeding history of each genotype to be traced through ICARDA records. The check varieties, PDL1 and PSL9, were released by ICAR-Indian Agricultural Research Institute (ICAR-IARI), of which PDL1 was released in collaboration with ICARDA in 2019. PDL1 is a small-seeded variety with an average yield of 983 kg ha^−1,^ whereas PSL9 is a bold-seeded variety with an average yield of 950 kg ha^−1,^ under saline condition.

### Experimental site and set up

The experiment was conducted at the Food Legumes Research Platform of ICARDA, Amlaha, Sehore, Madhya Pradesh, India that is located at 76.90°E and 23.12° N latitude with at an altitude of 498.77 m above sea level (ASL). The pot experiment and salinity imposition protocol were adapted following the methodology described by Joshi et al. (2023) and Kakar et al. (2019), with minor modifications. The study was performed under controlled polyhouse conditions using polyvinyl chloride (PVC) pots measuring 20.24 cm in diameter, 30.48 cm in height, with a capacity of 6.5 L. Each pot was layered with 500 g of gravel at the base to facilitate adequate drainage and filled with a homogenized soil mixture comprising black cotton soil and fine sand (particle size <0.3 mm) in a 3:1 ratio. The experimental soil was classified as black cotton soil with clayey texture, pH 7.8, organic carbon 0.62%, available nitrogen 225 kg ha^−1^, available phosphorus 17.5 kg ha^−1^, and available potassium 212.4 kg ha^−1^. The initial physicochemical properties of the potting medium included a near neutral to slightly alkaline pH (∼7.8) and low electrical conductivity (EC) of ∼0.225 dS m^−1^, indicating a non-saline baseline. Before sowing, the soil was uniformly amended with di-ammonium phosphate (DAP) @ 2 g kg^−1^ soil, thoroughly mixed in a 1:2 proportion with the soil to ensure adequate nutrient availability. The experimental pots were arranged in a completely randomized design (CRD) with three replications to minimize environmental variation, and pots were rotated periodically at regular intervals to minimize positional effects and ensure uniform exposure to environmental conditions.

### Growth conditions, and salt stress treatment

The experiment was conducted under controlled polyhouse conditions, where temperature was maintained at 22 °C ± 2 °C during the day and 18 °C ± 2 °C at night, with a relative humidity of 45% ± 5% and natural photoperiod conditions throughout the study. Lentil seeds were surface-sterilized with carbendazim 50 WP (Bavistin® 50 WP) at 2 g L^−1^ to prevent seed-borne fungal infections. Initially, ten seeds were sown per pot and subsequently thinned to five uniform and healthy seedlings per pot 1 week after emergence to ensure uniform plant density. Plants were grown under two treatment conditions, saline and non-saline (control) conditions. Salt stress was imposed at the 1-week seedling stage by irrigating pots with an 80 mM NaCl solution, prepared by dissolving a calculated quantity of analytical-grade NaCl in tap water corresponding to an EC of 8 dS m^−1^. To minimize osmotic shock, salinity was imposed gradually in a stepwise manner from the seventh to the 10th day after sowing. The target salinity level (ECe = 8 dS m^−1^) was achieved on the 10th day and maintained thereafter throughout the experiment. Salinity levels in the pots were monitored regularly using a conductivity meter, and excess salts were prevented from accumulating through periodic drainage. The pots were maintained near maximum field capacity throughout the experiment. The control and salt solution were replaced every 2 days. To ensure uniform and consistent salinity levels, the ECe of the potting medium was monitored on alternate days. For ECe determination, a 1:2 soil–water extract method was used. Briefly, 25 g of air-dried soil (<2 mm) was mixed with 50 mL distilled water, shaken mechanically for 1 h, and filtered through Whatman No. 1 filter paper. Electrical conductivity of the extract (EC1:2) was measured using a conductivity meter (EUTECH CON 2700, Singapore). The measured EC values were converted to saturated paste extract conductivity (ECe) using a conversion factor of 4.2.

### Observations on seedling morphological traits

Seedling morphological traits were recorded from 30-day-old seedlings grown under control and salinity treatments. The traits included shoot length (SL), root length (RL), total fresh weight (TFW), shoot fresh weight (SFW), and root fresh weight (RFW). Following fresh weight measurements, plant samples were oven-dried at 65 °C for at least 3 days until a constant weight was achieved. Dry biomass parameters, including total dry weight (TDW), shoot dry weight (SDW), and root dry weight (RDW), were subsequently recorded. Three randomly selected plants were sampled from each pot for recording observations under both control and salinity stress conditions. The measurements were taken individually from the selected plants within each pot, and the mean values were subsequently used for statistical analysis and data presentation.

### Salt stress response indices

Stress tolerance indices were calculated to effectively evaluate the responsiveness of lentil genotypes to salt stress. The cumulative stress tolerance index (CSTI) for each genotype was computed following the method described by Joshi et al. (2023). Initially, individual stress response indices were calculated for each measured seedling morphological parameter under control and stress conditions. These indices were then integrated to derive the CSTI, which represents the overall tolerance performance of a genotype across multiple traits. The CSTI was calculated as the sum of the individual trait-based stress indices, thereby providing a comprehensive measure of salinity tolerance:

$$\text{CSTI}=\frac{{\left(RLi\right)}_{s}\times {\left(RLi\right)}_{ns}}{{\left(RLns\right)}^{2}}+\frac{{\left(SLi\right)}_{s}\times {\left(SLi\right)}_{ns}}{{\left(SLns\right)}^{2}}+\frac{{\left(TFWi\right)}_{s}\times {\left(TFWi\right)}_{ns}}{{\left(TFW_{ns}\right)}^{2}}+\frac{{\left(RFWi\right)}_{s}\times {\left(RFWi\right)}_{ns}}{{\left(RFW_{ns}\right)}^{2}}+\frac{{\left(SFWi\right)}_{s}\times {\left(SFWi\right)}_{ns}}{{\left(SFW_{ns}\right)}^{2}}+\frac{{\left(TDWi\right)}_{s}\times {\left(TDWi\right)}_{ns}}{{\left(TDW_{ns}\right)}^{2}}+\frac{{\left(RDWi\right)}_{s}\times {\left(RDWi\right)}_{ns}}{{\left(RDW_{ns}\right)}^{2}}+\frac{{\left(SDWi\right)}_{s}\times {\left(SDWi\right)}_{ns}}{{\left(SDW_{ns}\right)}^{2}}$$

In Equation, (*X*i)s indicates the value of a given parameter of the *i*th genotype under stress condition and (*X*i)ns indicates the value of a given parameter of the *i*th genotype under non-stress condition. In equation denominators, *X*ns is the mean value of a given parameter of all genotypes evaluated under non-stress (control) condition.

Lentil genotypes were categorized into three response groups based on the CSTI values in relation to population mean of all measured parameters and their standard deviations (SD). Genotypes were classified as salt sensitive (mean CSTI +0.25 SD), moderately salt tolerant (mean CSTI + 0.25–1.25 s.d.), and salt tolerant (mean CSTI +>1.25 SD). To further visualize the distribution of lentil genotypes across tolerance classes, a density curve was drawn to indicate a continuous representation of variation from sensitive to tolerant groups.

### Statistical analysis

Descriptive statistics, including mean, range, SD, were calculated for all measured traits. Data obtained from the pot experiment were subjected to analysis of variance (ANOVA) using CRD under a two-factor arrangement, with genotype as the main factor and salinity treatment as a sub-factor and their interaction included in the model. The ANOVA was performed using the agricolae package in R (v4.4.2), and treatment means were compared using critical difference (CD) at a significance level of *p* ≤ 0.05. The coefficient of variation (CV) was also calculated to assess experimental precision. Pearson’s correlation coefficient among morphological traits was estimated and visualized using the *metan* package. Principal component analysis (PCA) was computed by *FactoMineR* and *factoextra* packages to identify key traits contributing to salinity tolerance and to explore multivariate relationships among genotypes. Additionally, box and whisker plots and hierarchical clustering were performed based on the morphological response variables and indices by using the basic R statistical environment (v4.4.2).

Different indices were calculated based on seed yield under salinity stress (S) and non-stress (NS) conditions as follows:

Salinity yield index, SYI = $\frac{{\left(Y_{i}\right)}_{ns}/{\left(Y_{i}\right)}_{s}}{\;\left(Y_{G}\right)ns/\left(Y_{G}\right)s\;}$ (Raman et al., 2012)

Stress tolerance index, STI = $\frac{{\left(Y_{i}\right)}_{ns}\times {\left(Y_{i}\right)}_{s}}{{\left(Y_{ns}\right)}^{2}}$ (Fernandez et al., 1992)

Stress susceptibility index, SSI = $\frac{{\left(Y_{i}\right)}_{ns}-{\left(Y_{i}\right)}_{s}}{Y_{ns}-Y_{s}}\times \frac{{\left(Y\right)}_{ns}}{{\left(Y_{i}\right)}^{ns}}$ (Fischer et al., 1978)

$$\text{Percent reduction in yield},\%R=\frac{{\left(Y_{i}\right)}_{ns}-{\left(Y_{i}\right)}_{s}}{{\left(Y_{i}\right)}^{ns}\;}\times 100$$

Where Y_i_ and Y_G_ denote the average seed yield of the genotype i and the overall geometric mean, respectively; S and NS denote salinity-stressed and non-stress conditions; and Y is the overall mean across all the genotypes.

### Field validation of lentil genotypes under saline and non-saline conditions

A field experiment was conducted during the winter season of 2024–25 at the ICAR–Central Soil Salinity Research Institute (ICAR-CSSRI), Karnal, Haryana, India (29°43′ N latitude, 76°58′ E longitude and 245 m above mean sea level) under two contrasting field conditions, namely non-saline (control) and saline. The baseline soil had a pH of approximately 7.8 and an initial ECe of 0.225 dS m^−1^. The soil was classified as a basalt-derived Vertisol with predominantly clay texture (∼58% clay), mainly composed of smectite (montmorillonite) minerals. These soils are characterized by high water-holding capacity, shrink–swell behaviour, and relatively low hydraulic conductivity under wet conditions. The soluble ionic composition of the soil extract included Na^+^ = 2.5 meq L^−1^, Cl^−^ = 3.0 meq L^−1^, Ca^2+^ = 8.0 meq L^−1^, and Mg^2+^ = 4.0 meq L^−1^. In addition, the sodium adsorption ratio (SAR) and exchangeable sodium percentage (ESP) were 1.01 (mmol L^−1^)^1/^ ^2^ and 1.5%, respectively. The saline field was characterised by a soil electrical conductivity of the saturation extract (ECe ≈7 dS m^−1^) and a soil pH of approximately 7.8. The climate of the region is subtropical to tropical (hot sub-humid). The present study included 12 lentil genotypes, including tolerant, moderately tolerant and one susceptible line, along with two checks. These genotypes were sown in early November 2024 with randomized block design (RBD) in 3 replications. Each experimental plot measured 1.2 m^2^ and consisted of two rows of 2 m length with a row-to-row spacing of 30 cm and plant-to-plant spacing of 5 cm, accommodating approximately 80 plants per plot. All experimental plots under saline and non-saline conditions were managed uniformly following the recommended agronomic practices for lentil cultivation. A basal fertilizer dose equivalent to 20 kg N ha^−1^ and 40 kg P_2_O_5_ ha^−1^ was applied through di-ammonium phosphate (DAP) at the time of sowing. Seed treatment was done with carbendazim 50 WP (Bavistin® 50 WP) at 2 g L^−1^ to prevent seed-borne fungal infections. Irrigation was applied uniformly under both saline and non-saline environments at critical crop growth stages, particularly during pre-flowering and pod development stages, to avoid moisture stress interference during crop growth and establishment. Necessary plant protection measures were adopted as and when required following recommendations for lentil cultivation to minimize biotic stress effects on genotype performance. Soil salinity levels in the saline field were periodically monitored at 7-14 days intervals through measurement of soil electrical conductivity of the saturation extract (ECe) and soil pH using a digital conductivity meter and pH meter following the saturated soil paste extract method described by Richards (1954). The saline field maintained an average ECe of approximately 7 dS m^−1^ with soil pH around 7.8 throughout the crop growth period to ensure consistent salinity stress conditions.

### Observation of morpho-phenological data

Data were recorded on key morpho-phenological and yield-related traits, including days to 50% flowering (DFF), days to maturity (DM), plant height (PH) (cm), number of branches per plant (NBP), number of pods per plant (NPP), 100-seed weight (g) (HSW), seed yield per five plants (SYFP) (g), biological yield per five plants (BYFP) (g), and seed yield (SY) (kg ha^−1^). One plot was considered as the experimental unit for statistical analysis, and total plot yield was recorded on a plot basis. In addition, yield-related observations were recorded from five randomly selected plants within each plot, and the yield per five plants was calculated by summing the yield of all five sampled plants. Yield data were recorded at physiological maturity from the entire plot and converted to kg ha^−1^ using plot size as the conversion factor.

Shoot and root samples were collected separately from plants grown under both control and saline field conditions. Samples were thoroughly rinsed three times with distilled water to remove surface contaminants, and oven-dried at 65 °C for 72 h until a constant weight was achieved. The dried shoot and root tissues were finely ground, and 0.5 g of each sample was digested using a diacid digestion (20 mL) using a mixture of nitric acid (HNO_3_) and perchloric acid (HClO_4_) in a 9:4 ratio on a hot-plate digestion system. The digested samples were diluted with double-distilled water, and the final volume was adjusted to 50 mL. After filtration through Whatman No. 42 filter paper, Na^+^ and K^+^ concentrations were determined using a flame photometer (Systronics Flame Photometer 128, SYSTRONICS, India) following the protocol described by Yadav et al., (2020) and Shekhawat et al. (2025).

### Gene expression analysis

The expression profiles of two key salinity-responsive genes, namely the *HKT* (*Lcu.2RBY.2g061250*) and NAC domain-containing protein-72, were analyzed in selected lentil genotypes showing contrasting responses to salt stress. The *HKT* gene, located on chromosome 2, was selected based on its strong association with salt tolerance in lentil, identified through a genome-wide association study conducted by Dissanayake et al. (2021). The NAC gene was selected based on its reported involvement in salt stress responses in lentil, as described by Singh D. et al. (2021). The study included five salt-tolerant genotype, namely X2018-482-S5, ILL7547, X2011S-89-23-S1, X2011S-91-77-2-S1, and ILL6819/ILL6207-S2, along with one salt-sensitive line (FLIP2014-083L-S9) and a tolerant check (PDL-1).

### Plant growth conditions and sample collection

Seeds of all lentil genotypes were surface sterilized using 12% sodium hypochlorite for 10 min and subsequently rinsed three times with distilled water. Thirty seeds per genotype were placed in Petri dishes lined with filter paper moistened with distilled water and allowed to germinate under controlled conditions. A hydroponic experiment was carried out in a climate-controlled plant growth chamber at the Plant Genomics Laboratory, ICARDA- FLRP, Amlaha, Sehore India. Environmental conditions were maintained at 22/18 °C (±2 °C) day/night temperature, a 10-h light/14-h dark photoperiod and relative humidity of approximately 45%. Seven-day-old uniform seedlings were transferred to a hydroponic system containing Hoagland nutrient solution following the standard protocol (Samineni et al., 2011; Singh et al., 2017). The experiment was conducted in three biological replications and arranged in two sets, one maintained under control conditions and the other subjected to salt stress. The hydroponic nutrient solution was supplemented with NaCl to achieve a final concentration of 100 mM. Following the method described by Hiz et al. (2014), NaCl was added stepwise at 50 mM increments over two consecutive days using Hoagland nutrient solution. This gradual salinity imposition approach minimized plasmolysis and osmotic injury in seedlings under salt stress conditions, as also reported by Shavrukov (2013). Leaf tissues were collected at 6 and 12 h after salt stress imposition, along with corresponding control samples. For each treatment and time point, three biological replicates were harvested, with each replicate consisting of pooled leaf tissue from three seedlings. Samples were immediately frozen in liquid nitrogen and kept at −80 °C for further analysis.

### Quantitative real-time polymerase chain reaction (qRT-PCR)

Total RNA was extracted from leaf tissues using the NucleoSpin RNA Kit (MACHEREY-NAGEL, Germany) following the manufacturer’s instructions. RNA concentration and purity were assessed using a NanoDrop ONE^c^ spectrophotometer (Thermo Fisher Scientific, United States). First-strand cDNA was synthesized from 2 μg of total RNA using the PrimeScript™ first Strand cDNA Synthesis Kit (Takara Bio USA, Inc., United States) according to the manufacturer’s protocol. The *HKT* gene (*Lcu.2RBY.2g061250*) coding DNA sequence (CDS) was retrieved from the KnowPulse JBrowse database (Ramsay et al., 2021; Yuan et al., 2021; https://knowpulse.usask.ca/jbrowse/Lens-culinaris/2), and gene-specific primers were designed using the NCBI Primer-BLAST tool (https://www.ncbi.nlm.nih.gov/tools/primer-blast/). Primer sequences for the *NAC* gene were adopted from Singh D. et al. (2021). Primers’ details are presented in Table 1. The primers were validated using method of Sen Gupta et al. (2017). The calibration curves for each primer pair were generated using five serial dilutions of cDNA to determine amplification efficiency, slope, and coefficient of determination (*R* ^2^). To confirm amplification specificity, a melting curve analysis was included at the end of the Quantitative real-time PCR (qRT-PCR) run, where a single sharp peak indicated specific amplification without primer-dimer formation or non-specific products. Amplification efficiencies were calculated as E = (10^-1/slope^-1) × 100 (Sen Gupta et al., 2017).

**TABLE 1:** Details of qRT-PCR primers.

Target gene	Primer sequence		References
LcActin	Forward	CCA​AAT​CAT​GTT​TGA​GGC​TTT​TAA	Sen Gupta et al. (2017)
	Reverse	GTG​AAA​GAA​CGG​CCT​GAA​TAG​C	
*HKT (Lcu.2RBY.2g061250)*	Forward	TTG​CAA​CAC​ATC​ATG​CCA​AAA​AC	​
	Reverse	TGA​TGT​GTT​GCA​AAG​ATG​TTG​C	
NAC domain-containing protein 72 (XP_004514350)	Forward	CGG​AAG​CCA​AAC​ACA​TGG​GA	Singh D. et al. (2021)
	Reverse	GAA​CCT​CTT​CTT​CCG​TGG​GG	

qRT-PCR was performed using a QuantStudio™ 5 Real-Time PCR System (Applied Biosystems, Thermo Fisher Scientific, United States). Each 20 μL reaction mixture contained 10 μL of TB Green® Premix Ex Taq™ (Takara Bio USA, Inc., United States), 0.8 μL each of forward and reverse primers, 0.4 μL of ROX reference dye, and 1 μL of cDNA template. Thermal cycling conditions included an initial denaturation at 95 °C for 5 min, followed by 40 cycles of denaturation at 95 °C for 20 s, annealing at 60 °C for 30 s, and extension at 72 °C for 10 s. A melt curve analysis was performed to confirm amplification specificity. Relative gene expression levels were calculated using the 2^−ΔΔCT^ method (Livak and Schmittgen, 2001). The lentil actin gene (*Lcu.2RBY.L011470.1*) was used as an internal reference for normalisation (Sen Gupta et al., 2017; Yuan et al., 2021). Each reaction was performed with three technical replicates per biological replicate, and results were expressed as mean ± standard error (SE). Statistical significance between treatments was assessed using a Student’s t-test at *p* ≤ 0.05.

## Results

### Effect of salinity stress on seedling growth and biomass

**FIGURE 1:**
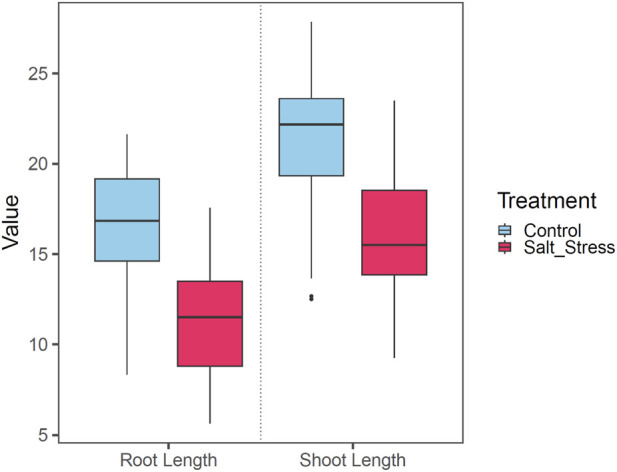
Box plots illustrating root length and shoot length, highlighting natural variation and the impact of salt stress on these morphological traits in lentil.

Salinity stress exerted a significant effect on seedling growth and biomass accumulation in lentil, with ANOVA revealing highly significant (*p* ≤ 0.001) effects of genotype, salinity and their interaction across the traits under control and salinity conditions (Table 2). However, SFW was non-significant for genotype and salinity interactions. The relatively low CV with the range of 3.21%–9.61% indicated good experimental precision (Table 3). Under salinity stress (8 dS m^−1^), a marked reduction in all seedling morphological traits was observed compared to control conditions. Mean SL decreased substantially, accompanied by a pronounced decline in root length, indicating that both above- and below-ground growth were adversely affected under salt stress. Under non-saline conditions, SL ranged from 12.5 cm (X2012S-171-17-S3) to 27.85 cm (X2018-482-S5), with a mean of 21.20 cm, whereas under salinity, the mean SL declined to 15.93 cm (∼24% reduction). Similarly, RL under control had a range from 8.33 cm to 21.63 cm with a mean of 16.45 cm, which decreased to 11.42 cm under salinity stress, accounting for ∼30% reduction (Figure 1; Table 4). However, the extent of reduction varied among genotypes, highlighting considerable genetic variability in salinity response. It was observed that, in the check PDL-1, the reduction due to salinity was less from 24.5 to 21.5cm and 19.23 to 17.5 cm in SL and RL, respectively. Similarly, less reduction (12%–15%) was also seen in tolerant genotype, *viz.*, X2011S-91-77-2-S1, ILL 7547 and PSL-9, reflecting better adaptability under salinity.

**TABLE 2:** Analysis of variance on seedling morphological traits across 50 lentil genotypes, treatments and their interaction.

S.No	Trait	DF	Genotype (MSS)	Salinity (MSS)	Genotype × salinity (MSS)	Error MSS	P-value
1	SL	49	65.203^***^	2,110.887^***^	7.683^***^	1.326	4.9E-88
2	RL	49	53.574^***^	1904.768^***^	11.49^***^	1.032	3.6E-90
3	SFW	49	0.541^***^	16.457^***^	0.132^NS^	0.113	1.5E-15
4	RFW	49	0.113^***^	2.101^***^	0.029^***^	0.006	3.5E-57
5	TFW	49	0.886^***^	30.318^***^	0.213^**^	0.117	1.3E-25
6	SDW	49	0.091^***^	1.798^***^	0.026^***^	0.004	6.1E-67
7	RDW	49	0.013^***^	0.373^***^	0.005^***^	0.001	6.3E-125
8	TDW	49	0.147^***^	3.806^***^	0.041^***^	0.004	9.1E-84

MSS, mean sum of square; SL, shoot length; RL, root length; SFW, shoot fresh weigh; RFW, Root fresh weight, TFW, total fresh weight; SDW, shoot dry weight; RDW, root dry weight; and TDW, total dry weight. ^**^P < 0.01; ^***^P < 0.001

**TABLE 3:** Descriptive statistics for seedling morphological traits in 50 lentil genotypes under saline and non-saline condition.

​	SL		RL		SFW		RFW		TFW		SDW		RDW		TDW	
	C	S	C	S	C	S	C	S	C	S	C	S	C	S	C	S
Mean	21.2	15.94	16.46	11.43	1.28	0.82	0.39	0.22	1.67	1.03	0.34	0.19	0.12	0.05	0.46	0.24
Max	27.85	23.5	21.64	17.56	2.33	1.82	0.72	0.49	2.73	2.2	0.87	0.54	0.22	0.15	1.04	0.65
Min	12.5	9.25	8.34	5.65	0.68	0.35	0.07	0.06	0.89	0.48	0.13	0.07	0.01	0.01	0.15	0.08
Range	15.35	14.25	13.31	11.91	1.65	1.48	0.65	0.43	1.84	1.73	0.75	0.47	0.21	0.15	0.89	0.57
CV	8.27	7.46	6.36	8.63	7.53	8.12	3.21	3.87	3.46	3.40	8.67	9.61	4.75	3.87	4.99	3.87
CD	1.81	1.92	1.7	1.6	0.74	0.23	0.13	0.11	0.74	0.26	0.11	0.08	0.03	0.01	0.12	0.08
SEm	0.65	0.69	0.61	0.57	0.27	0.09	0.05	0.04	0.27	0.1	0.04	0.03	0.01	0.01	0.04	0.03
SEd	0.92	0.97	0.86	0.81	0.38	0.12	0.07	0.06	0.38	0.13	0.06	0.04	0.02	0.01	0.06	0.04

C, control; S, salinity stress; SL, shoot length; RL, root length; SFW, shoot fresh weigh; RFW, Root fresh weight, TFW, total fresh weight; SDW, shoot dry weight; RDW, root dry weight; and TDW, Total dry weight. CV, coefficient of variation; CD, critical difference; SEM, standard error of mean; SED, standard error of difference.

**TABLE 4:** Mean performance of 50 lentil genotypes for seedling morphological traits under control and salinity stress conditions.

S.No	Genotypes	SL (cm)		RL (cm)		SFW (g)		RFW (g)		TFW (g)		SDW (g)		RDW (g)		TDW (g)	
		C	S	C	S	C	S	C	S	C	S	C	S	C	S	C	S
1	X2012S-109-S13	19.67	12.6	14.67	9.62	0.95	0.51	0.28	0.16	1.23	0.67	0.21	0.09	0.093	0.028	0.31	0.12
2	X2012S-146-S15	18.33	10.25	18.17	7.58	1.09	0.34	0.31	0.13	1.40	0.47	0.14	0.06	0.120	0.017	0.26	0.08
3	X2012S-148-S15	13.66	9.25	9.46	8.65	0.78	0.43	0.16	0.06	0.94	0.49	0.12	0.08	0.020	0.006	0.14	0.08
4	X2012S-171-17-S3	12.50	9.76	8.33	5.65	0.84	0.56	0.12	0.08	0.96	0.64	0.15	0.10	0.010	0.006	0.16	0.11
5	ILL7978/ILWL118-S11-S22	21.67	14.76	16.67	13.5	1.47	0.90	0.43	0.18	1.90	1.08	0.37	0.16	0.100	0.014	0.47	0.18
6	ILL10829/ILWL130-S-5	19.33	13.43	13.17	11.56	0.91	0.53	0.22	0.15	1.13	0.68	0.20	0.10	0.063	0.02	0.26	0.12
7	ILL18029/ILWL130-S-50-S4	23.67	14.50	20.53	17.5	1.67	0.92	0.72	0.13	2.39	1.05	0.38	0.17	0.220	0.018	0.60	0.19
8	FLIP2014-032L-S1-S45	15.67	11.50	13.33	9.56	0.98	0.57	0.27	0.16	1.25	0.73	0.17	0.07	0.080	0.025	0.25	0.10
9	X2013S-17-20-3-S1	20.00	16.25	17.87	10.65	1.08	0.70	0.45	0.27	1.53	0.97	0.27	0.14	0.060	0.061	0.33	0.20
10	FLIP2014-028L-S1	23.00	14.50	14.77	11.56	1.18	0.68	0.19	0.09	1.37	0.77	0.29	0.12	0.080	0.007	0.37	0.13
11	X2011S-89-23-S1	22.75	17.35	19.17	15.65	1.52	0.92	0.61	0.48	2.13	1.40	0.48	0.39	0.120	0.114	0.60	0.50
12	FLIP2014-083L-S9	18.33	12.50	16.33	8.56	1.13	0.67	0.28	0.12	1.41	0.79	0.35	0.12	0.085	0.01	0.43	0.13
13	X2011S-91-77-2-S1	24.67	21.35	20.70	17.56	1.65	0.93	0.62	0.46	2.27	1.39	0.51	0.37	0.190	0.138	0.70	0.51
14	X2012S-56252-2-S1-S6	22.75	15.45	21.63	13.58	1.11	0.65	0.68	0.24	1.79	0.89	0.30	0.12	0.190	0.042	0.49	0.16
15	X2011S-148-S1-S12	19.50	15.67	17.90	12.50	0.68	0.56	0.47	0.33	1.15	0.89	0.21	0.18	0.141	0.099	0.35	0.28
16	ILL6778/ILL5480-S3-S4	23.06	19.50	16.47	11.65	1.24	0.95	0.36	0.28	1.60	1.23	0.33	0.27	0.150	0.084	0.48	0.36
17	ILL6002/LIRL21-50-1-17-5-S3	22.75	13.50	19.13	8.50	1.34	0.69	0.58	0.25	1.92	0.94	0.41	0.12	0.170	0.035	0.58	0.16
18	ILL18029/ILWL130-S57-S45	23.30	16.50	17.57	6.67	1.35	0.86	0.53	0.07	1.88	0.93	0.39	0.16	0.166	0.006	0.55	0.16
19	2008S-41121-04-S2	23.33	17.50	16.77	11.50	1.09	0.72	0.43	0.32	1.52	1.04	0.33	0.23	0.180	0.096	0.51	0.33
20	ILL813/4605/1-2-S1-S87	24.35	18.50	20.13	13.75	2.14	0.73	0.58	0.22	2.72	0.95	0.67	0.13	0.220	0.026	0.89	0.16
21	59018461/2-4-S1	21.50	16.33	16.33	11.36	1.21	0.82	0.38	0.16	1.59	0.98	0.28	0.15	0.114	0.048	0.39	0.20
22	X211S-61-8-S1	16.67	13.85	13.20	9.32	0.86	0.61	0.23	0.11	1.09	0.72	0.17	0.11	0.061	0.01	0.23	0.12
23	2011S-89-23-S4	21.75	18.54	17.73	11.25	1.25	0.97	0.68	0.21	1.93	1.18	0.30	0.18	0.200	0.023	0.50	0.20
24	X2012S-183-S9	18.50	14.65	16.30	11.50	2.05	0.73	0.36	0.22	2.41	0.95	0.32	0.13	0.160	0.026	0.48	0.16
25	ILL 7547	24.50	21.50	19.17	16.33	1.68	1.35	0.48	0.41	2.16	1.76	0.47	0.35	0.210	0.123	0.68	0.47
26	FLIP2014-086L-S1	19.56	15.50	16.90	13.50	1.28	0.91	0.39	0.12	1.67	1.03	0.28	0.17	0.110	0.017	0.39	0.18
27	ILL18029/ILWL130-S-68	23.65	18.67	18.40	16.67	1.32	1.01	0.57	0.48	1.89	1.49	0.40	0.33	0.140	0.124	0.54	0.45
28	X2018-482-S5	27.85	23.5	19.57	14.6	2.23	1.82	0.43	0.38	2.66	2.20	0.84	0.53	0.190	0.114	1.03	0.64
29	ILL8006/ILWL62-S25-S5	18.50	16.5	12.33	9.85	0.98	0.77	0.26	0.19	1.24	0.96	0.25	0.22	0.090	0.09	0.34	0.31
30	ILL10847-S5-S15	22.50	16.25	16.33	12.5	1.21	0.78	0.18	0.08	1.39	0.86	0.42	0.14	0.055	0.007	0.48	0.15
31	ILL6002/ILWL4402-S1	13.67	10.67	11.33	8.42	0.86	0.57	0.13	0.1	0.99	0.67	0.16	0.10	0.030	0.008	0.19	0.11
32	ILL8461/ILL8006/4-6-S1	20.75	17.67	17.53	12.35	0.86	0.69	0.45	0.37	1.31	1.06	0.22	0.19	0.135	0.12	0.35	0.31
33	ILL7537/ILL8006-S4-S25	18.50	13.85	13.23	9.60	0.78	0.48	0.31	0.20	1.09	0.68	0.14	0.09	0.093	0.024	0.23	0.11
34	2010S96134-2-S1-S8	19.33	14.50	15.19	12.50	1.08	0.71	0.26	0.19	1.34	0.90	0.28	0.13	0.079	0.027	0.36	0.16
35	2009S-83103-16M-S2	22.50	18.70	20.27	17.45	1.14	0.85	0.52	0.47	1.66	1.32	0.33	0.28	0.157	0.141	0.49	0.42
36	2009S-96501-S5	22.50	13.50	18.03	13.50	1.09	0.55	0.56	0.09	1.65	0.64	0.28	0.10	0.182	0.006	0.46	0.11
37	ILL6819/ILL6207-S2	25.67	21.80	17.20	15.35	1.35	1.09	0.51	0.46	1.86	1.55	0.45	0.38	0.150	0.148	0.60	0.53
38	ILL18029/ILWL130-S155-S12	23.50	11.75	13.07	9.50	1.84	0.82	0.19	0.12	2.03	0.94	0.63	0.15	0.098	0.016	0.73	0.17
39	ILL6002/7716/4-4-S2	24.67	16.50	15.67	8.37	1.33	0.89	0.22	0.09	1.55	0.98	0.34	0.16	0.050	0.006	0.39	0.17
40	X2012S-1-S4-S10	26.15	18.50	19.17	9.25	2.33	1.08	0.38	0.13	2.71	1.21	0.86	0.20	0.115	0.017	0.98	0.21
41	FLIP2014-116L-S10	23.86	15.50	18.73	7.65	1.65	0.97	0.38	0.15	2.03	1.12	0.30	0.20	0.140	0.018	0.44	0.22
42	ILL7531/8461/3-1-S2	19.67	14.65	12.83	6.35	1.21	0.80	0.21	0.11	1.42	0.91	0.32	0.15	0.034	0.008	0.35	0.15
43	ILL6002/LIRL21-50-1-17-4-S3	21.85	19.67	16.03	12.50	1.35	1.02	0.33	0.22	1.68	1.24	0.35	0.18	0.140	0.096	0.49	0.28
44	LIRL-21-187-S1-S28	24.86	18.65	21.23	11.67	1.79	0.82	0.66	0.26	2.45	1.08	0.43	0.15	0.177	0.048	0.60	0.20
45	X2013S-20-3-25-S1	22.67	14.85	19.50	8.52	1.23	0.71	0.39	0.17	1.62	0.88	0.37	0.13	0.100	0.016	0.47	0.14
46	ILL18029/ILWL130-S-56	12.67	9.87	8.70	5.66	0.82	0.64	0.07	0.06	0.89	0.70	0.14	0.12	0.010	0.006	0.15	0.12
47	FLIP 2014-062L-S3-S2	21.33	18.67	14.60	9.30	1.24	0.99	0.19	0.07	1.43	1.06	0.23	0.19	0.096	0.008	0.32	0.20
48	X2013S-19-16-S14	18.57	14.35	12.07	6.50	0.82	0.57	0.13	0.08	0.95	0.65	0.18	0.10	0.020	0.006	0.20	0.11
49	PSL-9	25.50	21.67	19.90	16.65	1.48	1.18	0.54	0.49	2.02	1.67	0.36	0.29	0.163	0.127	0.53	0.41
50	PDL-1	24.50	21.50	19.23	17.50	1.45	1.52	0.49	0.45	1.94	1.97	0.64	0.48	0.180	0.135	0.82	0.61
​	Mean	21.20	15.93	16.45	11.42	1.28	0.81	0.38	0.22	1.66	1.03	0.34	0.19	0.12	0.05	0.46	0.23

C, control; S, salinity stress C, control; S, salinity stress; SL, shoot length; RL, root length; SFW, shoot fresh weigh; RFW, Root fresh weight, TFW, total fresh weight; SDW, shoot dry weight; RDW, root dry weight; and TDW, total dry weight.

**FIGURE 2:**
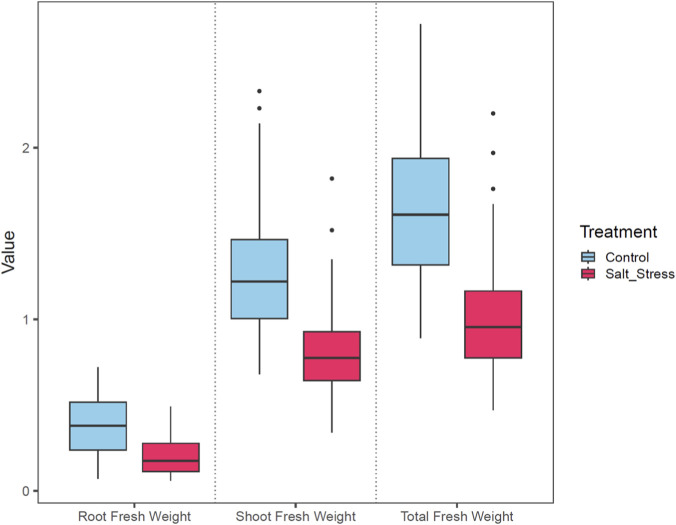
Box plots for root fresh weight, shoot fresh weight, and total fresh weight, showing natural variation and the effect of salt stress on seedling morphological parameters in lentil.

Substantial variation was observed among lentil genotypes for shoot, root and total biomass under both control and salinity conditions. Under control conditions, SFW ranged from 0.68 g to 2.33 g, with a mean of 1.28 g, which declined by ∼36% under salinity stress (mean: 0.81 g). A similar trend was observed for RFW and total fresh weight, with the deduction of 42.1% and 37.9%, respectively (Figure 2). Notably, several genotypes, including PDL-1, ILL 7547, X2018-482-S5, PSL-9, ILL6819/ILL6207-S2, and ILL18029/ILWL130-S-68, exhibited markedly lower reduction (around 20% and below) in fresh biomass under salinity, indicating enhanced tolerance.

**FIGURE 3:**
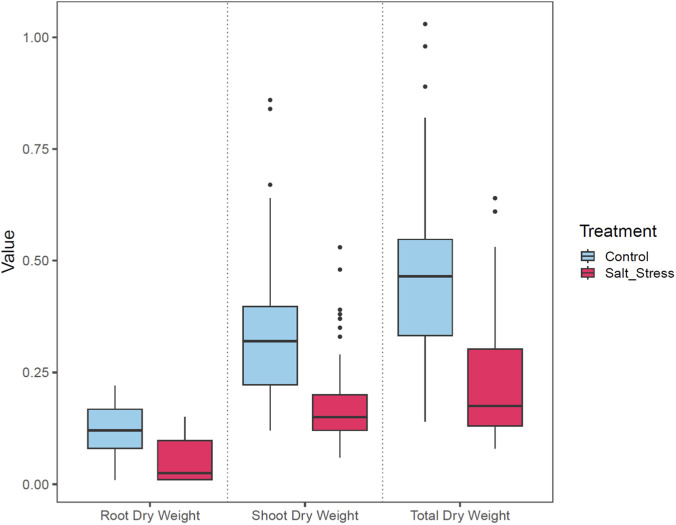
Box plots representing root dry weight, shoot dry weight, and total dry weight, illustrating the extent of natural variation among lentil genotypes as well as the influence of salt stress on these key morphological parameters.

Dry biomass was significantly reduced under salinity stress. SDW ranged from 0.12 to 0.86 g under control conditions with a mean of 0.34 g, but under salinity stress, from 0.06 to 0.53 g with a mean of 0.19 g, leading to a reduction of ∼44% under salinity. RDW and TDW declined by ∼58% (from 0.12 to 0.05 g) and ∼50% (from 0.46 to 0.23 g), respectively, under salinity compared to the control. Among the genotypes, ILL6819/ILL6207-S2 was less impacted by salinity with the lowest reduction in SDW (15.6%), RDW (1.3%) and TDW (11.7%), demonstrating superior biomass retention under salinity (Figure 3).

### Classification of lentil genotypes based on stress tolerance indices

**FIGURE 4:**
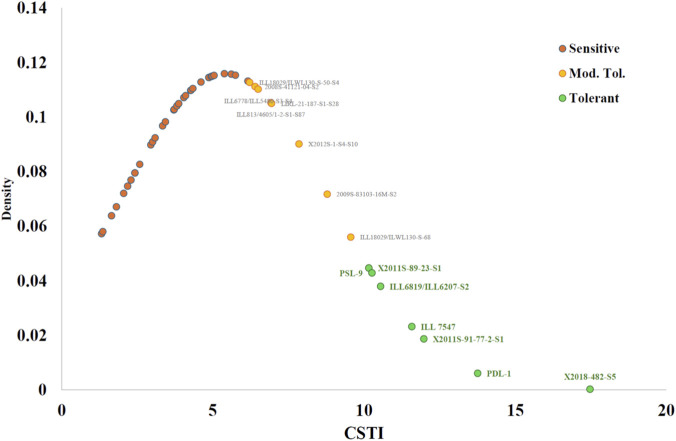
Density distribution of CSTI values showing classification of lentil genotypes into sensitive, moderately tolerant, and tolerant groups.

**FIGURE 5:**
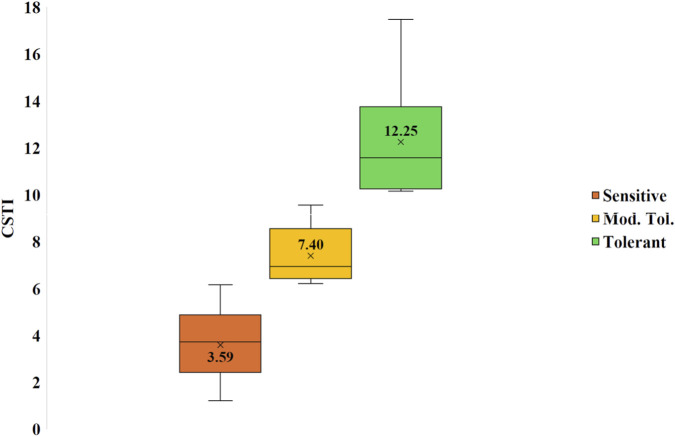
Box plot of CSTI values illustrating the distribution and variability among lentil genotypes, with clear separation into sensitive, moderately tolerant, and tolerant groups.

The cumulative stress tolerance index (CSTI) proved effective in differentiating lentil genotypes based on their salinity response (Figure 4; Table 4). Based on CSTI values relative to the population mean and standard deviation, genotypes were classified into salt-sensitive, moderately tolerant and salt-tolerant, with mean CSTI values of 3.79, 7.4 and 12.25, respectively (Figure 5). A majority of the genotypes were categorized as sensitive, which accounted for 70% (35 genotypes) under study. Similarly, eight genotypes (16%) were considered as moderately salt-tolerant with the CSIT value between 6.2 and 9.7. A total of seven genotypes with CSIT value more than 9.7, *viz.*, X2011S-89-23-S1, PSL-9, ILL6819/ILL6207-S2, ILL 7547, X2011S-91-77-2-S1, PDL-1 and X2018-482-S5 were considered as tolerant genotypes. The density distribution of CSTI values indicated a continuous variation across genotypes, with a clear separation between tolerant and sensitive groups.

### Correlation among the traits and CSTI

**FIGURE 6:**
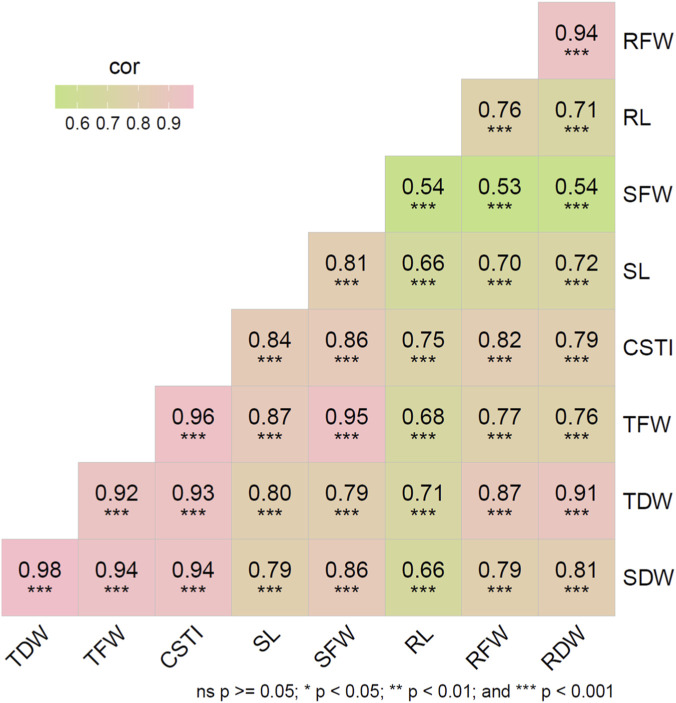
Correlation matrix showing relationships among seedling morphological traits and CSTI under salt stress conditions in lentil.

Correlation analysis revealed strong and highly significant positive associations (*p* < 0.001) among shoot and root traits, indicating coordinated regulation of growth under salinity stress (Figure 6). Biomass-related traits showed particularly strong interrelationship with each other (*r* = 0.92–0.98). SL and SFW also exhibited highly significant positive correlations with both fresh and dry weight of root and total biomass traits. Root traits, including RFW and RDW, showed a strong positive correlation (*r =* 0.94***) and were similarly correlated with other traits. Further, CSTI showed a highly significant positive correlation with all the traits, indicating that improved growth performance contributed to enhanced salinity tolerance. CSTI was strongly associated with TDW, TFW, and SDW, with r value greater than 0.93 (p < 0.0001) and also with SL (*r* = 0.84***) and SFW (*r* = 0.75***). However, CSTI showed moderate to strong positive correlations with RFW (*r* = 0.82***) and RL (*r* = 0.53***).

### Multivariate analysis

**FIGURE 7:**
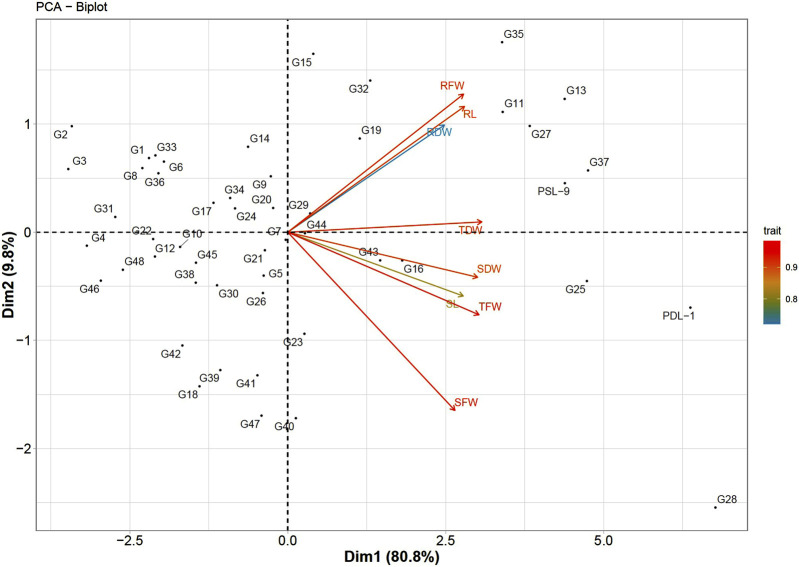
Principal component analysis (PCA) biplot illustrating the distribution of lentil genotypes and their association with seedling morphological traits under salt stress conditions.

Principal component analysis (PCA) based on eight seedling morphological traits revealed that the first two components explained 90.7% of the total variation (PC1: 80.9%; PC2: 9.8%) (Figure 7). The PCA indicated that root traits, particularly RL, RFW, and RDW, were the most important contributors, followed by biomass-related traits (TFW, TDW, SDW, SFW), and SL. The PCA further revealed strong associations among traits, where RL, RFW, and RDW were closely related, while TFW, TDW, SDW, and SFW formed another closely associated group. SL also showed a positive association with biomass traits. The PCA biplot clearly separated salt-tolerant and sensitive genotypes along PC1, with tolerant genotypes clustering with checks PDL-1 and PSL-9, while sensitive genotypes grouped on the opposite side. This highlights the effectiveness of PCA in discriminating genotypes based on salinity tolerance.

**FIGURE 8:**
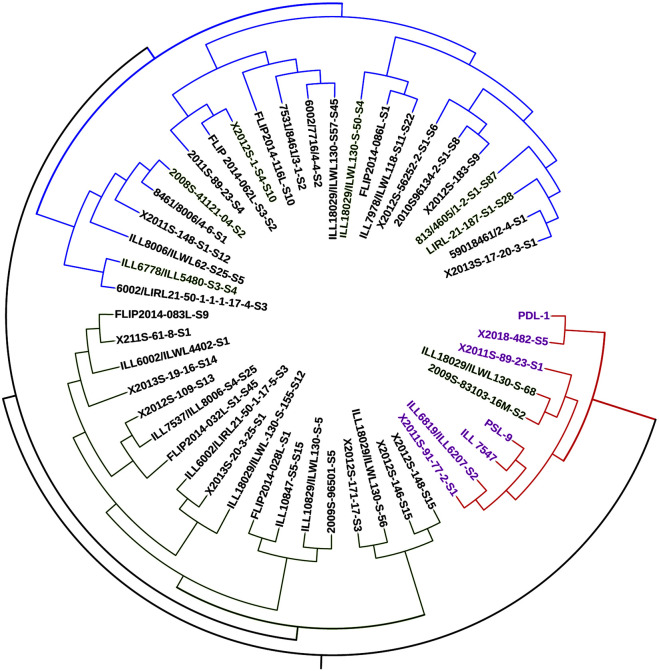
Clustering of 50 lentil genotypes into three distinct groups based on seedling morphological traits under salt stress conditions, highlighting genetic diversity and variation in stress response.

Cluster analysis further grouped 50 lentil genotypes into three distinct clusters corresponding to their tolerance levels, reinforcing the classification obtained through CSTI (Figure 8). Cluster-I comprised nine genotypes, predominantly represented by salt-tolerant genotypes, including PDL-1, PSL-9, ILL7547, X2011S-91-77-2-S1, X2018-482-S5, X2011S-89-23-S1, and ILL6819/ILL6207-S2, which performed well under salinity stress conditions. However, two moderately salt-tolerant genotypes, ILL18029/ILWL130-S-68 and 2009S-83103-16M-S2, were also grouped within Cluster-I. In contrast, Cluster-II comprised 23 genotypes, which were mainly categorized as moderately salt-tolerant. However, this cluster also included several salt-sensitive genotypes, indicating a mixed grouping pattern and suggesting intermediate responses to salinity stress. Additionally, Cluster-III comprised 18 genotypes, which were the worst performers under salinity stress conditions and were classified as highly salt sensitive.

### Field validation of lentil genotypes for salinity tolerance

A field trial under salinity was conducted with 12 genotypes selected from the previous experiment to evaluate the transferability of the result in real field situations. ANOVA revealed highly significant effects (*p* ≤ 0.001) of genotype, salinity, and their interaction for most phenological, growth, yield, and ionic traits, indicating differential genotype responses under field conditions (Table 5). The GE was significant for most traits, including days to flowering, plant height, number of pods, yield and biological yield, and all root and shoot ionic traits, indicating differential response of genotypes to salinity under the field. However, the interaction effect was non-significant for the number of branches and 100-seed weight, suggesting stable expression under control and salinity treatment.

**TABLE 5:** Classification of 50 lentil genotypes using the cumulative salt tolerance index (CSTI) of seedling morpho-physiological parameters.

Sensitive genotype	CSTI (1.22-6.16)	Moderately tolerant genotype	CSTI (6.22-9.56)	Tolerant genotype	CSTI (10.16-17.48)
X2012S-148-S15	1.22	ILL18029/ILWL130-S-50-S4	6.22	X2011S-89-23-S1	10.16
X2012S-171-17-S3	1.32	2008S-41121-04-S2	6.4	PSL-9	10.26
ILL18029/ILWL130-S-56	1.36	ILL6778/ILL5480-S3-S4	6.5	ILL6819/ILL6207-S2	10.55
ILL6002/ILWL4402-S1	1.65	ILL813/4605/1-2-S1-S87	6.92	ILL 7547	11.58
X2013S-19-16-S14	1.81	LIRL-21-187-S1-S28	6.94	X2011S-91-77-2-S1	11.98
X2012S-146-S15	2.05	X2012S-1-S4-S10	7.85	PDL-1	13.76
X211S-61-8-S1	2.18	2009S-83103-16M-S2	8.78	X2018-482-S5	17.48
FLIP2014-032L-S1-S45	2.29	ILL18029/ILWL130-S-68	9.56	​	​
ILL7537/ILL8006-S4-S25	2.42	​	​	​	​
ILL10829/ILWL130-S-5	2.42	​	​	​	​
X2012S-109-S13	2.58	​	​	​	​
FLIP2014-083L-S9	2.95	​	​	​	​
ILL7531/8461/3-1-S2	3.01	​	​	​	​
FLIP2014-028L-S1	3.09	​	​	​	​
2009S-96501-S5	3.34	​	​	​	​
2010S96134-2-S1-S8	3.43	​	​	​	​
FLIP 2014-062L-S3-S2	3.71	​	​	​	​
ILL10847-S5-S15	3.72	​	​	​	​
ILL6002/7716/4-4-S2	3.81	​	​	​	​
X2013S-20-3-25-S1	3.87	​	​	​	​
ILL8006/ILWL62-S25-S5	4.04	​	​	​	​
ILL18029/ILWL130-S57-S45	4.1	​	​	​	​
FLIP2014-086L-S1	4.27	​	​	​	​
X2013S-17-20-3-S1	4.28	​	​	​	​
59018461/2-4-S1	4.34	​	​	​	​
ILL18029/ILWL130-S155-S12	4.61	​	​	​	​
X2012S-183-S9	4.87	​	​	​	​
ILL7978/ILWL118-S11-S22	4.87	​	​	​	​
ILL6002/LIRL21-50-1-17-5-S3	4.95	​	​	​	​
FLIP2014-116L-S10	4.98	​	​	​	​
X2011S-148-S1-S12	5.04	​	​	​	​
X2012S-56252-2-S1-S6	5.38	​	​	​	​
2011S-89-23-S4	5.61	​	​	​	​
ILL8461/ILL8006/4-6-S1	5.75	​	​	​	​
ILL6002/LIRL21-50-1-17-4-S3	6.16	​	​	​	​
70%	​	16%	​	14%	​

Salinity stress resulted in an overall reduction in agro-morphological and yield traits across genotypes (Table 6). The tolerant checks PDL-1 and PSL-9 recorded seed yields of 1,153 kg ha^−1^ and 1,106 kg ha^−1^, respectively. X2011S-91-77-2-S1 recorded the highest seed yield under salinity (1,386 kg ha^−1^), exceeding PDL-1 by 20.2% and PSL-9 by 25.3%, along with a high biological yield and yield per five plants. Similarly, ILL6819/ILL6207-S2 produced 1,190 kg ha^−1^ and outperformed PSL-9 by 7.6%, with yield comparable to PDL-1, while ILL 7547 and X2018-482-S5 recorded statistically at par yield with the checks, indicating stable performance under salt stress. Tolerant genotypes, particularly X2011S-91-77-2-S1 and ILL6819/ILL6207-S2, also maintained higher biological yield and pod number under stress compared to both checks. In contrast, only marginal reductions were observed for days to flowering and maturity, across genotypes, including checks under salinity stress. Plant height and branching were generally reduced under salinity across the genotypes and checks. However, salt-tolerant genotypes like ILL 7547, X2011S-89-23-S1 and ILL6819/ILL6207-S2 retained higher branching under stress comparable to that of checks (Table 7).

**TABLE 6:** Analysis of variance for agro-physiological and biochemical traits in 12 accessions of lentil under control and salinity.

S.No	Traits	DF	Genotype (MSS)	Salinity (MSS)	Genotype x salinity (MSS)	Error MSS	P-value
1	Days to flowering (Days)	11	397.42***	385.33***	12.33***	2.17	1.12413E-19
2	Time to maturity (Days)	11	49.61***	36.75***	3.57*	1.55	2.58345E-11
3	Plant Height (cm)	11	91.78***	459.42***	6.39*	3.67	3.31855E-10
4	No. of branches per plant	11	14.4***	102.38***	0.53^ns^	0.93	3.94626E-08
5	No. of pods per plant	11	9,077.95***	39606.03***	2,590.47***	365.60	3.61908E-10
6	100 Seed wt. (g)	11	0.2***	0.33***	0.02^ns^	0.02	3.65875E-06
7	Yield/5 plant (g)	11	463.98***	6,603.52***	215.66**	51.14	5.38674E-06
8	Bilogical Yield/5 plants (gm)	11	4,208.87***	103509.19***	2,401.55***	274.46	4.43912E-08
9	Seed yield (kg/ha)	11	167050.38***	3946138.83***	53271.57***	9,236.56	8.87267E-09
10	Root Na (mg g^−1^ dry weight)	11	286.97***	12927.14***	265.37***	3.84	2.58953E-15
11	Root K (mg g ^−^ ^1^ dry weight)	11	43.68***	5,683.28***	94.76***	3.34	1.98406E-07
12	Root Na/K	11	301.21***	22771.86***	301.31***	2.05	1.32002E-18
13	Shoot Na (mg g ^−^ ^1^ dry weight)	11	71.14***	333.91***	115.97***	1.34	1.09749E-13
14	Shoot K (mg g ^−^ ^1^ dry weight)	11	46.06***	21744.33***	105.81**	3.07	5.54694E-08
15	Shoot Na/K	11	6.11***	96.05***	6.09***	0.11	5.10385E-14

MSS, mean sum of square. ^*^P < 0.05; ^**^P < 0.01; ^***^P < 0.001.

**TABLE 7:** Agro-physiological and biochemical data of 12 accessions of lentil under control and salinity stress condition

Trait	Env	FLIP2014-028L-S1	ILL813/4605/1-2-S1-S87	ILL6778/ILL5480-S3-S4	X2012S-1-S4-S10	ILL18029/ILWL130-S-68	ILL 7547	X2011S-89-23-S1	X2011S-91-77-2-S1	ILL6819/ILL6207-S2	X2018-482-S5	PSL-9	PDL-1	CV%	CD	SEM
Days to flowering (Days)	C	67.0	79.5	64.0	89.0	64.5	86.5	89.0	86.0	84.5	62.5	88.0	87.5	1.90	3.31	1.06
	S	63.0	71.5	62.0	81.0	62.5	81.5	83.0	81.5	81.5	60.0	78.5	74.0	2.03	3.27	1.05
Time to maturity (Days)	C	125.0	127.0	128.0	128.5	130.0	129.0	131.5	125.0	127.5	123.0	130.5	134.5	0.92	2.61	0.84
	S	125.0	125.0	125.5	123.0	130.5	128.0	127.5	122.0	126.5	120.5	129.5	135.5	1.05	2.93	0.94
Plant Height (cm)	C	23.3	39.6	30.0	40.8	36.1	35.8	36.0	37.8	39.2	34.6	40.3	39.3	4.56	3.62	1.16
	S	18.6	33.0	23.7	31.6	29.4	32.4	32.0	31.7	26.7	29.1	36.3	34.1	7.24	4.76	1.53
No. of Branches per plant	C	7.1	9.5	7.9	11.9	8.7	12.5	12.8	11.7	12.3	9.8	12.2	10.5	8.66	2.01	0.65
	S	4.4	5.9	4.8	7.8	6.1	9.4	9.9	9.0	8.3	7.7	9.7	8.9	12.05	2.03	0.65
No. of Pods per plant	C	187.8	236.1	205.1	205.5	102.1	256.8	237.8	257.9	196.7	245.2	227.8	229	11.24	53.35	17.14
	S	18.5	100.4	129.2	169.1	82.4	187.7	216.2	221.4	182.3	166.1	214.2	210.9	8.38	29.19	9.38
100 Seed wt. (g)	C	2.2	2.0	2.0	1.7	2.3	2.4	1.8	2.3	2.3	2.3	2.3	1.9	8.67	0.41	0.13
	S	2.1	1.9	1.9	1.6	1.7	2.3	1.7	2.2	2.1	2.1	2.0	1.8	4.89	0.21	0.07
Yield/5 plant (g)	C	62.0	72.5	41.5	37.0	34.0	61.5	66.0	65.0	69.0	58.5	48.5	47.5	15.08	18.34	5.89
	S	13.0	18.0	23.0	28.5	9.0	38.5	44.5	39.5	47.5	40.5	39.5	40.0	19.23	13.46	4.32
Biological Yield/5 plants (g)	C	248.5	190.5	208.5	218.0	183.5	197.5	280.0	254.0	242.0	148.5	226.5	203.5	9.16	43.70	14.04
	S	58.0	68.0	127.5	103.5	93.0	114.5	115.5	178.5	208.0	105.0	142.0	173.0	8.44	23.00	7.39
Seed yield (kg/ha)	C	1,323	1,253	1,390	1,453	1,523	1,500	1,466	1,643	1,613	1,323	1,565	1,511	7.61	245.28	78.8
	S	386	453	775	690	620	1,028	920	1,190	1,386	975	1,106	1,153	7.71	151.07	48.54
Root Na^+^ (mg g^−1^ dry weight)	C	13.2	13.0	13.2	13.9	13.3	12.1	13.3	12.4	12.1	12.8	12.6	12.0	9.70	2.73	0.88
	S	75.4	77.9	47.7	52.6	54.8	35.1	37.0	37.6	29.1	33.4	34.2	32.8	4.95	4.97	1.60
Root K^+^ (mg g^−1^ dry weight)	C	0.34	0.33	0.30	0.35	0.30	0.31	0.37	0.36	0.32	0.36	0.34	0.31	5.75	4.89	1.57
	S	6.7	7.2	11.2	10.1	9.9	22.2	15.5	18.3	23.1	25.3	26.2	27.1	7.70	2.86	0.92
Root Na^+^/K^+^	C	0.36	0.33	0.30	0.33	0.37	0.36	0.36	0.32	0.34	0.32	0.31	0.34	7.58	0.06	0.02
	S	11.28	10.87	4.30	5.28	5.58	1.58	2.39	2.07	1.26	1.31	1.31	1.21	4.27	4.13	1.33
Shoot Na^+^ (mg g^−1^ dry weight)	C	15.6	15.2	16.7	17.6	11.3	14.5	17.6	15.8	13.6	16.3	10.2	10.0	8.28	2.65	0.85
	S	62.4	66.9	49.0	74.2	57.2	34.5	38.7	35.1	25.5	28.9	27.4	26.8	5.60	2.44	0.78
Shoot K^+^ (mg g^−1^ dry weight)	C	52.1	50.2	47.1	49.5	49.7	46.0	42.4	44.1	47.9	40.9	46.6	42.4	5.24	5.37	1.73
	S	7.6	10.9	14.1	13.4	12.7	17.6	15.7	18.9	33.6	32.8	29.8	30.5	17.01	1.51	0.49
Shoot Na^+^/K^+^	C	0.30	0.30	0.35	0.36	0.23	0.32	0.42	0.36	0.28	0.40	0.22	0.24	11.06	0.08	0.02
	S	8.20	6.21	3.49	5.55	4.52	1.98	2.49	1.86	0.76	0.88	0.92	0.88	14.36	0.99	0.32

C, Control; S, Salinity ECe 7 dS/m.

### Assessment of salinity tolerance using stress indices

Salinity tolerance of lentil genotypes was further evaluated using percent yield reduction (%R) and stress indices (Table 8). Tolerant genotypes such as ILL6819/ILL6207-S2, X2018-482-S5, X2011S-91-77-2-S1 and ILL7547 exhibited marginal yield reduction (15.51%–32.25%) upon salinity stress, similar to that of checks, PDL-1 and PSL-9. Among these, ILL6819/ILL6207-S2 showed superior performance with the lowest SSI (0.36) and the highest STI (1.04). In contrast, FLIP2014-028L-S1 was identified as the most susceptible genotype, exhibiting the highest yield reduction (64.02%). A shift in genotype response was observed under field conditions, where ILL813/4605/1-2-S1-S87, despite showing a moderately tolerant response at the seedling stage, exhibited high susceptibility at the reproductive stage, as reflected by greater yield reduction (54.66%), lower STI (0.26), and higher SSI (1.63). In contrast, other moderately tolerant genotypes (ILL6778/ILL5480-S3-S4, X2012S-1-S4-S10, and ILL18029/ILWL130-S-68) maintained intermediate performance, reflecting partial but less stable tolerance under salinity stress.

**TABLE 8:** Seed yield and indices for salinity tolerance in 12 lentil genotypes.

S.No	Genotypes	Seed yield (kg/ha)		%R	SYI	STI	SSI
		Control	Salinity stress				
1	FLIP2014-028L-S1	1,323	386	64.02	2.08	0.24	1.81
2	ILL813/4605/1-2-S1-S87	1,253	453	54.66	1.68	0.26	1.63
3	ILL6778/ILL5480-S3-S4	1,390	775	42.02	1.09	0.50	1.13
4	X2012S-1-S4-S10	1,453	690	52.13	1.28	0.47	1.34
5	ILL18029/ILWL130-S-68	1,523	620	61.70	1.49	0.44	1.51
6	ILL 7547	1,500	1,028	32.25	0.89	0.72	0.80
7	X2011S-89-23-S1	1,466	920	37.31	0.97	0.63	0.95
8	X2011S-91-77-2-S1	1,643	1,190	30.95	0.84	0.91	0.70
9	ILL6819/ILL6207-S2	1,613	1,386	15.51	0.71	1.04	0.36
10	X2018-482-S5	1,323	975	23.78	0.83	0.60	0.67
11	PSL-9	1,565	1,106	31.36	0.86	0.81	0.75
12	PDL-1	1,511	1,153	24.46	0.80	0.81	0.60

%R, percentage reduction in yield; SYI, salinity yield index; STI, stress tolerance index; SSI, stress susceptibility index.

### Ion accumulation and ionic homeostasis in the shoot and root

Under salinity stress, checks and tolerant genotypes exhibited lower shoot Na^+^ and Na^+^/K^+^ ratios. Compared with the checks PDL-1 (root Na^+^/K^+^ = 1.21; shoot Na^+^/K^+^ = 0.88) and PSL-9 (root Na^+^/K^+^ = 1.31; shoot Na^+^/K^+^ = 0.92), X2011S-91-77-2-S1 maintained a lower shoot Na^+^/K^+^ ratio (0.76), along with reduced shoot Na^+^ (25.5 mg g^−1^) and relatively high shoot K^+^ (33.6 mg g^−1^). Similarly, ILL6819/ILL6207-S2, X2018-482-S5, and X2011S-89-23-S1 showed improved ionic balance compared to the susceptible line FLIP2014-028L-S1. Genotypes with higher or comparable yield to the checks consistently showed better Na^+^ exclusion and lower Na^+^/K^+^ ratio in both roots and shoots. Considering overall performance, X2011S-91-77-2-S1, followed by ILL6819/ILL6207-S2 and X2018-482-S5, demonstrated superior ionic homeostasis under salinity (Table 7).

### Correlation among physiological traits

**FIGURE 9:**
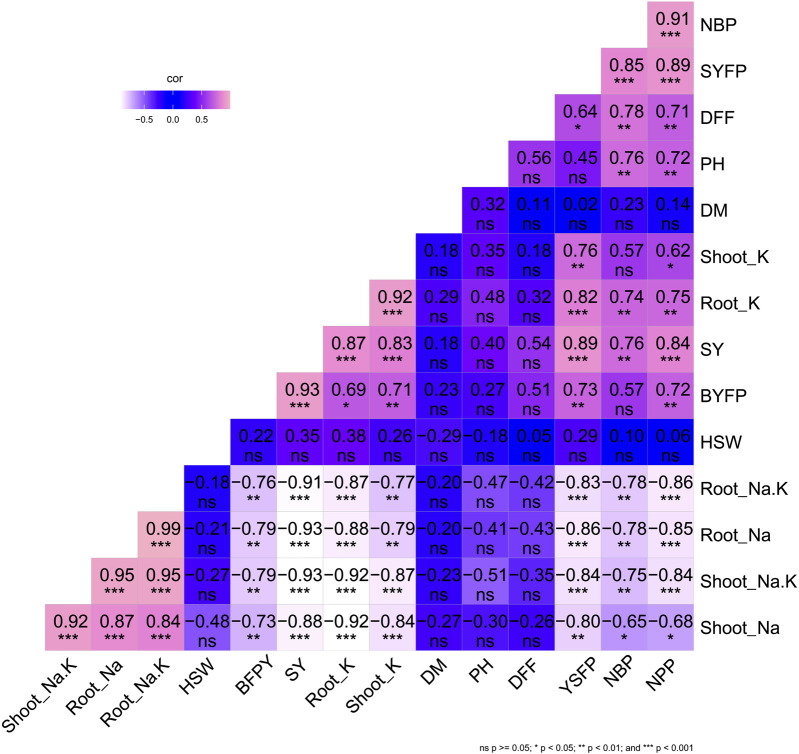
Correlation matrix showing relationships among agro-morphological traits and ion content under field salt stress conditions in lentil.

Correlation analysis revealed the strong association of physiological and ionic traits with seed yield under salinity conditions in the field (Figure 9). SY showed a significant positive correlation with BY (*r* = 0.93^***^), SYFP (*r* = 0.89^***^), and NPP (*r* = 0.84^***^). Among ionic traits, SY exhibited strong negative correlations with both shoot Na^+^ (*r* = −0.88^***^), root Na^+^ (*r* = −0.93^***^), shoot Na^+^/K^+^ ratio (*r* = −0.93^***^), and root Na^+^/K^+^ ratio (*r* = −0.91^***^). Conversely, positive correlations were observed with root K^+^ (*r* = 0.82^***^) and shoot K+ (*r* = 0.74^**^). A significant positive correlation between root and shoot K^+^ (*r* = 0.92^***^) further highlights the role of K^+^ retention in salinity tolerance. However, traits like DFF, PH, DM and HSW showed non-significant correlation with ionic traits.

### Expression dynamics of key salinity tolerance genes

**FIGURE 10:**
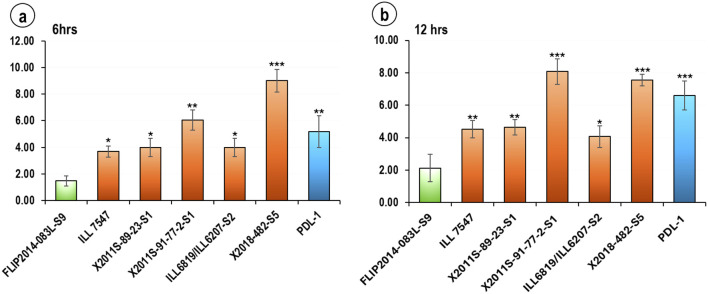
Expression profiling of *NAC* gene in response to salinity stress. **(a)** 6 h after salt treatment; **(b)** 12 h after salt treatment. As per Student’s t-test, ^***^ *p* < 0.001, ^**^ *p* < 0.01 and ^*^ *p* < 0.05.

**FIGURE 11:**
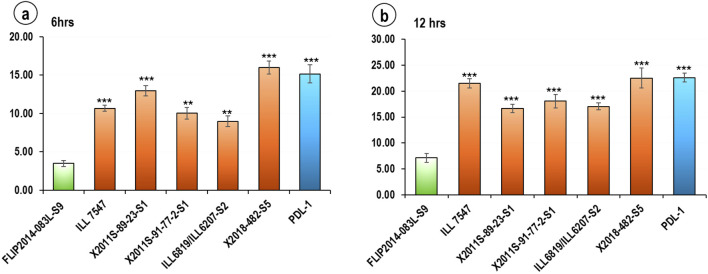
Expression profiling of *HKT* gene in response to salinity stress. **(a)** 6 h after salt treatment; **(b)** 12 h after salt treatment. As per Student’s t-test, ^***^ *p* < 0.001, ^**^ *p* < 0.01 and ^*^ *p* < 0.05.

Gene expression profiling revealed significant upregulation of *HKT* and *NAC domain-containing protein-72* genes in salt-tolerant lentil genotypes and the tolerant check (PDL-1) compared with the salt-sensitive check (FLIP2014-083L-S9) under salinity stress. The *NAC domain-containing protein-72* gene showed rapid induction at 6 h in tolerant genotypes, followed by sustained or genotype-dependent modulation at 12 h (Figure 10). Tolerant genotypes showed markedly higher expression than the sensitive check at both time points. Peak expression at 12 h was observed in X2011S-91-77-2-S1, followed by X2018-482-S5 and PDL-1 (Figure 10). In contrast, the *HKT* gene displayed the most pronounced upregulation, indicating its central role in ion homeostasis under salinity (Figure 11). At 6 h, the sensitive check showed a relatively low expression level, while tolerant genotypes exhibited strong induction, with the highest levels in X2018-482-S5, closely followed by the tolerant check PDL-1 and X2011S-89-23-S1. Expression further increased at 12 h across all tolerant genotypes, with maximum upregulation in PDL-1 and X2018-482-S5, followed by ILL 7547 (Figure 11). Overall, tolerant genotypes exhibited consistently higher expression than the sensitive check, with a clear temporal increase from 6 h to 12 h.

## Discussion

Lentil, like most grain legumes, is inherently sensitive to salinity stress, particularly during germination, early seedling establishment, and the reproductive phase, which are considered the most critical phases determining crop establishment and final productivity (Singh et al., 2017). Salinity-induced inhibition of germination, root elongation, and seedling vigour has been widely documented in lentil and is primarily attributed to osmotic imbalance and ion toxicity, which collectively impair water uptake, cellular expansion, and metabolic activity (Singh et al., 2017; Foti et al., 2019; Shekhawat et al., 2025). Consequently, efficient seedling establishment plays a pivotal role in improving lentil performance under saline environments. Given the strong stage-specific sensitivity, early-stage phenotyping has emerged as a practicable and reliable strategy for identifying salt-tolerant genotypes with stable performance across environments. However, field-based screening for salinity tolerance remains challenging due to spatial heterogeneity of soil salinity, environmental variability, and the need for multi-location and multi-year evaluations, which collectively limit precision and increase resource demands (Kakar et al., 2019). In this context, controlled environment screening techniques, particularly pot-based assays, offer a robust alternative by enabling uniform stress imposition and reliable evaluation of genotypes. Such approaches have been successfully employed across multiple crops, including lentil (Dissanayake et al., 2021; Shekhawat et al., 2025), rice (Kakar et al., 2019; Barman et al., 2025), and chickpea (Joshi et al., 2023) demonstrating their effectiveness in dissecting physiological variability and facilitating the identification of salt-tolerant genotypes under standardized conditions.

### Relative performance of lentil genotypes under salt stress

Salinity-induced growth inhibition in lentil is primarily governed by osmotic stress and ion toxicity, which together restrict water uptake, disrupt cellular expansion, and impair metabolic activity (Singh et al., 2017; Foti et al., 2019; Panuccio et al., 2022). The pronounced reduction in seedling growth observed in the present study is therefore consistent with the well-established effects of salinity on plant physiological processes. The present study highlighted the existence of substantial genetic variability for salinity tolerance among the evaluated lentil genotypes, which is essential for effective selection and breeding (Kumawat et al., 2017; Dissanayake et al., 2021; Joshi et al., 2023). The reduction in shoot (∼24%) and root length (∼30%) under salinity stress reflects the inhibitory effects of salt on cell elongation and division due to reduced water uptake and ion toxicity. The relatively greater sensitivity of roots compared to shoots can be attributed to their direct exposure to saline conditions and their role as the primary sites of stress perception and ion entry (Skliros et al., 2018; Paul et al., 2023). This differential response highlights the importance of early root-mediated signaling and adaptive responses in determining overall plant tolerance.

Similar reductions in vegetative growth under salinity have been widely reported in lentil and other legumes (Singh et al., 2017; Panuccio et al., 2022). The superior performance of certain tolerant genotypes (X2018-482-S5, ILL 7547, X2011S-91-77-2-S1, ILL6819/ILL6207-S2, and X2011S-89-23-S1 and salt tolerance checks PDL-1 and PSL-9) under salinity stress suggests the presence of coordinated tolerance mechanisms rather than isolated trait advantages. This improved performance may be attributed to efficient osmotic adjustment, Na^+^ exclusion, and maintenance of cellular homeostasis and preservation of metabolic function (Ali et al., 2024; Sallam et al., 2026). Such mechanisms are critical for sustaining cell turgor, enzymatic activity, and growth under stress conditions (Shivani et al., 2025). In contrast, sensitive genotypes are more prone to ionic imbalance and metabolic disruption, resulting in greater growth inhibition.

Biomass-related traits, including fresh and dry weights, showed even greater reductions under salinity stress, particularly root dry weight and total dry weight, indicating a deeper impact on carbon assimilation, photosynthesis, and biomass partitioning (Panuccio et al., 2022). These findings are consistent with earlier studies showing that salinity stress impairs photosynthesis through stomatal closure, chlorophyll degradation, and disruption of electron transport chains, ultimately limiting assimilate production and allocation (Bandeoğlu et al., 2004; Atta et al., 2023). Nevertheless, certain genotypes, such as ILL6819/ILL6207-S2 and X2018-482-S5, maintained relatively higher biomass under stress, suggesting the presence of efficient tolerance mechanisms such as better osmotic regulation, ion homeostasis, and enhanced antioxidant activity. The greater responsiveness of root-related traits, compared to shoot traits, further emphasizes the central role of root system architecture in salinity adaptation. Roots play a critical role in regulating ion uptake, maintaining water balance, and preventing excessive Na^+^ accumulation in aerial parts (Singh et al., 2017; Dissanayake et al., 2021). This functional significance explains why root traits are often more reliable indicators of salinity tolerance and have been consistently identified as key selection criteria in lentil and other crops.

### Concordance of CSTI and multivariate analysis for classification of lentil genotypes

Salinity tolerance has previously been categorized using integrative indices such as PCA, salt stress response indices (SSRI) and CSTI across multiple crops, including corn (Wijewardana et al., 2016), rice (Kakar et al., 2019), and chickpea (Joshi et al., 2023) to identify genotypes and key parameters. In the present study, approximately 14% of genotypes were identified as tolerant, with X2018-482-S5, X2011S-91-77-2-S1, ILL7547, ILL6819/ILL6207-S2, and X2011S-89-23-S1 emerging as top performers. Therefore, CSTI-based classification provided a reliable framework for distinguishing genotypes based on their ability to sustain performance under stress relative to non-stress conditions. Unlike single-trait evaluation, CSTI captures the combined effects of growth and stress response, thereby enabling the identification of genotypes with both high productivity and stress resilience. The strong positive association of CSTI with biomass-related traits, particularly TDW, TFW, and SDW, further highlight the central role of growth maintenance in salinity tolerance. This relationship suggests that the capacity to preserve biomass under stress is not merely a consequence of tolerance but a defining functional attribute of it. Such responses are closely linked to higher photosynthetic efficiency and biomass accumulation under stress (Bouallegue et al., 2025).

The close correspondence between PCA and CSTI outcomes indicates the reliability and complementarity of both approaches in classifying lentil genotypes for salinity tolerance. PCA proved to be an effective multivariate tool for identifying the major sources of variations and the relative contribution of traits under salinity stress (Negrao et al., 2017), whereas CSTI integrates these trait responses into a single index reflecting overall performance. The convergence of these methods therefore strengthens confidence in the classification of genotypes and the identification of key adaptive traits. Notably, both approaches consistently emphasized the greater relevance of root-associated parameters compared to shoot traits in explaining variability under salinity stress. This highlighted the functional significance of root systems in mediating stress adaptation through the regulation of water uptake and ion transport. The prominence of root traits in multivariate analyses has been similarly reported in lentil and other crops (Fernandez, 1992; Raman et al., 2012; Kakar et al., 2019; Joshi et al., 2023), and their higher loadings under saline conditions further support their utility as reliable selection criteria (Panuccio et al., 2022; Shekhawat et al., 2025). The concordance between PCA and CSTI, and clustering analyses, also indicates that morphological trait-based grouping reflects underlying physiological responses to salinity. However, the occurrence of mixed genotypic grouping in Cluster-I and Cluster-II further suggests that salinity tolerance in lentil is a complex quantitative trait governed by multiple physiological and genetic factors (Singh et al., 2017; Ali et al., 2024).

### Field validation

The field validation highlights that salinity tolerance in lentil is a context-dependent trait, shaped by both developmental stage and environmental variability. The highly significant effects of genotype, salinity, and GE interaction across most traits indicate substantial genetic variation for salt response, reinforcing the limitation of relying solely on controlled-environment screening. Such interactions are widely reported in legumes and emphasize that tolerance expressed under controlled environments may not always translate to field performance (Samineni et al., 2011; Singh et al., 2017; Farooq et al., 2017; Nadeem et al., 2019; Turner et al., 2022; Atta et al., 2023).

The pattern of yield response suggests that salinity primarily constrains productivity through its effects on growth and reproductive processes rather than through major shifts in phenology. This indicates that stress-induced limitations in carbon assimilation, nutrient balance, and assimilate partitioning play a more decisive role than shifts in crop duration. Similar mechanisms have been reported in lentil and other legumes, where yield losses under salinity are largely attributed to reduced reproductive efficiency, particularly through limitations in pod formation and seed set (Farooq et al., 2017; Foti et al., 2019; Panuccio et al., 2022; Shekhawat et al., 2025). Comparable responses in chickpea further support the notion that reproductive processes are more vulnerable to salinity than vegetative growth (Vadez et al., 2007; Samineni et al., 2011; Khan et al., 2017). The consistent performance of certain genotypes (X2011S-91-77-2-S1, followed by ILL6819/ILL6207-S2 and X2018-482-S5) under field salinity conditions indicates their capacity to maintain functional stability across environments. Such stability likely arises from the integration of multiple tolerance mechanisms, enabling these genotypes to sustain growth and reproductive output under ionic and osmotic stress. This aligns with previous findings in lentil, where similar genotype-dependent differences have been reported, with tolerant accessions showing better preservation of growth, photosynthetic performance, and seed yield under salt stress (Singh et al., 2017; Dissanayake et al., 2021; Panuccio et al., 2022). The identification of genotypes with stable performance across controlled and field environments strengthens their potential utility in breeding programs targeting saline agro-ecosystems.

The physiological basis of this stability is closely linked to the regulation of ion homeostasis. Maintenance of a low Na^+^/K^+^ ratio, achieved through restricted Na^+^ accumulation and sustained K^+^ uptake, is a key determinant of salinity tolerance. This pattern is central to salinity tolerance because K^+^ supports enzyme activity, osmotic regulation, and photosynthetic metabolism, whereas excess Na^+^ disrupts cellular function and competes with K^+^ uptake (Munns and Tester, 2008; Farooq et al., 2017; Singh D. et al., 2021; Dissanayake et al., 2021). The strong negative correlations of seed yield with ionic balance strongly support the central role of ionic regulation as a mechanistic driver of field-level tolerance.

### Molecular regulation of salt tolerance in lentil

The transcriptional responses observed in this study point to a coordinated regulatory framework underlying salinity tolerance in lentil. The rapid induction of the *NAC* gene in tolerant genotypes suggests its role in early stress perception and signal transduction. NAC transcription factors are widely recognized as key regulators of abiotic stress tolerance, modulating downstream gene networks associated with osmotic adjustment, detoxification, and secondary cell wall formation for cellular protection (Yamaguchi and Demura, 2010; Nuruzzaman et al., 2013; Shao et al., 2015; Singh D. et al., 2021). Their differential expression between tolerant and sensitive genotypes further supports their role as key modulators of stress adaptability in lentil (Singh D. et al., 2021; Goudarzi et al., 2024). In contrast to the regulatory role of *NAC*, the sustained induction of *HKT* gene highlights its direct involvement in maintaining ionic balance under salinity stress. This is highly significant because the lentil GWAS study by Dissanayake et al. (2021) identified *Lcu.2RBY.2g061250,* a high-affinity potassium transporter, as a high-priority salt-tolerance candidate on chromosome 2. HKT transporters are central to Na^+^ exclusion and recirculation mechanisms, preventing excessive Na^+^ accumulation in shoots and thereby preserving cellular function, which is one of the most important physiological determinants of salt tolerance. Efficient regulation of Na^+^/K^+^ homeostasis is widely recognized as a critical determinant of salinity tolerance, linking molecular activity to physiological performance. The combined activation of regulatory (*NAC*) and functional (*HKT*) components suggests an integrated tolerance mechanism in which early stress signalling is tightly coupled with downstream ionic regulation. Such coordination enables tolerant genotypes to rapidly perceive stress and implement adaptive responses that sustain cellular homeostasis. This mechanistic linkage provides a molecular explanation for the observed physiological stability under salinity. Importantly, the alignment between gene expression patterns and field performance indicates that these molecular responses are not merely transient or context-specific but translate into agronomically relevant tolerance. Genotypes exhibiting stronger induction of these genes are likely to possess enhanced capacity for stress perception and ion regulation, ultimately supporting improved growth and yield under saline conditions.

## Conclusion

The present study demonstrated substantial genetic variability for salinity tolerance in lentil at both seedling and field levels, highlighting the complex and multi-dimensional nature of salt stress response. Salinity significantly reduced growth, biomass, and yield-related traits; however, tolerant genotypes maintained comparatively higher performance under stress conditions. The integration of pot culture screening with field validation proved effective in identifying stable and high-performing genotypes under real saline environments. The identified superior genotypes represent valuable genetic resources for breeding programs aimed at developing salt-tolerant cultivars. These findings emphasize that effective selection should integrate early-stage screening, field validation, and physiological and molecular characterization to ensure stable performance under salinity.

## Data Availability

The original contributions presented in the study are included in the article/supplementary material, further inquiries can be directed to the corresponding authors.
